# Simulations as a mode of clinical training in healthcare professions: A scoping review to guide planning in speech-language pathology and audiology during the COVID-19 pandemic and beyond

**DOI:** 10.4102/sajcd.v69i2.905

**Published:** 2022-08-02

**Authors:** Nabeelah Nagdee, Ben Sebothoma, Milka Madahana, Katijah Khoza-Shangase, Nomfundo Moroe

**Affiliations:** 1 Department of Audiology, Faculty of Humanities, University of the Witwatersrand, Johannesburg, South Africa; 2 School of Engineering, University of the Witwatersrand, Johannesburg, South Africa

**Keywords:** audiology, healthcare professionals, clinical training, COVID-19, simulations, speech-language pathology

## Abstract

**Background:**

Simulation plays an important role as an alternative method for training of students, particularly in health education. As a result of coronavirus disease 2019 (COVID-19) restrictions of face-to-face interactions, traditional teaching methods have been disrupted, increasing the need for alternative methods to supplement modes of student clinical training in healthcare programmes.

**Objectives:**

The scoping review aimed to determine what has been documented about simulation as a mode of clinical training in healthcare professions (HCPs) in order to guide speech-language pathology and audiology (SLP&A) professions during the COVID-19 pandemic and beyond.

**Method:**

A scoping review was conducted. Electronic bibliographic databases including Science Direct, PubMed, Scopus MEDLINE, ProQuest, Google Scholar and WorldCat were searched to identify peer reviewed publications, published in English, between January 2011 and December 2021, and related to the use of simulation in HCPs clinical training programmes.

**Results:**

A total of 32 articles met the inclusion criteria for this scoping review. Three themes emerged when reviewing the studies: (1) face-to-face simulations as a mode of clinical training, (2) virtual reality simulation and telesimulation as modes of clinical training and (3) simulation as a complementary mode of clinical training. Evidence suggests that whilst simulations are cost-effective, accessible and efficacious as clinical training modes, they need to be combined with other modes of training such as the traditional clinical training to yield better learning outcomes.

**Conclusions:**

Current findings highlight the role and value of simulation as a clinical training mode during COVID-19 and beyond. However, there are aspects that need to be considered to ensure that this mode of clinical training is effective, with endorsement and regulations by the SLP&A Professional Board of the Health Professions Council of South Africa (HPCSA). Simulations need to be complemented with traditional clinical training methods. In the context of SLP&A, particularly in low- and middle-income countries (LMICs), simulation can be used to better prepare students for their clinical placement where clinical training platforms are limited and where simulation combined with teletraining or telesupervision can be utilised to increase access to training.

## Introduction

Simulations may be defined as scenarios that are artificially created to study or experience something that is possible in real life (Al-Elq, 2010; Datta et al., 2012). They have been used as a training strategy in high-risk professions like aviation, merchant marine, military and nuclear energy to enhance judgement and problem-solving skills, which lead to improved safety outcomes (Pham, 2019). Additionally, simulation has been found to be a cost-effective and less labour-intensive training method in these professions (Allerton, 2010).

To meet the growing demand for speech-language pathologists and audiologists, international and national universities that offer speech-language pathology and audiology (SLP&A) programmes were obliged to increase the intake of students into these programmes. However, this increased intake of students may have resulted in overburdening training platforms and university staff members who accommodate and facilitate SLP&A programmes (McAllister & Lincoln, 2004; O’Beirne, Kelly-Campbell, & Welch, 2020). This increase in student numbers may result in increased expenses to run SLP&A programmes as compared to non-clinical programmes, as a result of the demands of clinical training over and above the academic programme. Studies that have explored the effects of an increase in nursing student numbers on clinical placements have found that high student numbers place significant pressure on healthcare professional (HCP) clinical educators to teach, supervise and nurture the students towards clinical competency (Richardson & Claman, 2014; Roberts, Kaak, & Rolley, 2019). The same can be argued for the SLP&A programmes.

In addition to the high student-to-staff ratio, there is a clinical training platform challenge where there is a shortage of clinical placement sites, which may result in fewer learning opportunities and an increase in number of HCP students competing for supervision and access to the same patients (Feingold, Calaluce, & Kallen, 2004; Lambton, 2008). Because placing HCP students at appropriate clinical placements that support their learning is challenging, it may result in them feeling overwhelmed, less competent and demotivated to integrate and be involved in their clinical placements. This limitation in their training may hamper them from achieving the necessary core clinical competencies – knowledge, skills and attitudes – that are the intended learning outcomes at these placements (Griffin, McLeod, Francis, & Brown, 2016). These challenges and their effects may be exacerbated in HCP students’ clinical training programmes in low- and middle-income countries (LMICs) such as South Africa, because of the documented resource constraints (Akhlaq, McKinstry, Muhammad, & Sheikh, 2016; Khoza-Shangase & Mophosho, 2021; Pillay, Tiwari, Kathard, & Chikte, 2020).

Furthermore, the American Speech-Language-Hearing Association (ASHA n.d.) has formulated guidelines pertaining to the use of simulations in clinical practical courses accredited by the Council for Academic Accreditation in Audiology and Speech-Language Pathology (CFCC) (2016). Simulations, in the context of clinical practicals, are viewed as an alternative means of clinical training whereby students are able to develop their clinical knowledge and skills (ASHA n.d.). Furthermore, because of resource constraints that negatively affect clinical training of SLP&A students, the Council for Clinical Certification (2016) in the United States of America has allowed SLP&A students to obtain 75 hours of direct clinical contact hours when they engage in simulations.

The coronavirus disease 2019 (COVID-19) pandemic has additionally undoubtedly added to the aforementioned challenges by disrupting the clinical training in HCPs, where clinical training activities had to either be cancelled, paused or postponed (Haridy, Abdalla, Kaisarly, & Gezawi, 2021), globally. Furthermore, because of the social distancing regulations, clinical environments such as hospitals, which are traditional clinical training sites, needed to make adjustments, including limiting the presence of hospital staff and students and cancelling services considered to be non-essential to reduce patient numbers – in order to adhere to the regulations (Giordano, Cipollaro, Migliorini, & Maffulli, 2021). In South Africa, speech-language pathology outpatient services were suspended, and speech-language therapists took on additional roles such as conducting COVID-19 screenings which were part of the public health emergency priority for the National Department of Health (Adams, Seedat, Coutts, & Kater, 2020). These changes in functioning and emergency extensions to the scopes of practice may have resulted in HCP students not receiving sufficient clinical practical experiences to develop the necessary knowledge, skills and attitudes for clinical practice in their scopes of practice. Additionally, the interruption in the clinical training of HCP students caused them to experience psychosocial effects of COVID-19 such as anxiety because of uncertainty about the world (Giordano et al., 2021), as well as fear of contracting the virus and/or infecting others at their clinical placement sites (Rainford, 2021).

All these challenges highlight the need for innovative modes of providing clinical training during public health emergencies, such as during the COVID-19 pandemic, as well as beyond. Technological advances embedded within telehealth have been documented to be valuable to overcome these challenges, even as part of hybrid approaches to clinical training (Khoza-Shangase, Moroe, & Neille, 2021). Technological advances including the fourth industrial revolution have significant potential to overcome the challenges faced by healthcare education, such as mitigating the risk of compromising patients’ safety when HCP students learn about patient interactions and procedures on real patients (Guze, 2015). Furthermore, incorporating technology as a means of clinical training in healthcare education may support such a training shift towards competency-based education, as it facilitates basic knowledge acquisition, improves clinical skills and enhances decision-making. Simulation, specifically, allows for the fulfilment of educational goals such as the provision of feedback, repetitive practice, flexibility and the capturing of clinical variability (Guze, 2015).

Therefore, in light of the benefits of simulation training, the resource constraints that may affect HCP students’ learning and achievement of the core clinical competencies and the impact that COVID-19 has had on traditional clinical training of student HCPs, this scoping review was aimed at exploring published evidence on simulations as a mode of clinical training in HCPs, to plan for SLP&A training during the COVID-19 pandemic and beyond.

## Methodology

The researchers utilised the framework outlined by Arksey and O’Malley (2005) when conducting the scoping review, following five stages: (1) identifying the research question, (2) identifying relevant publications, (3) selecting studies to be included, (4) extracting and charting the data as well as (5) collating, summarising and reporting the results.

Additionally, the authors followed Levac, Colquhoun and O’Brien’s (2010) recommendation of using a research team when conducting a scoping review. The research team comprised of five researchers with experience in planning and supervising SLP&A student clinical training. They agreed on the broad research question to be addressed by the scoping review and the overall study protocol including the search terms and the databases to be searched.

### Research question

This scoping review sought to answer the following research question: ‘what has been documented about simulation as a mode of clinical training in HCPs?’ This research question was prompted by the need for alternative or supplemental modes of traditional clinical training to account for clinical training challenges encountered by SLP&A programmes during the COVID-19 pandemic. Findings from this scoping review will guide clinical training planning in SLP&A during the COVID-19 pandemic and beyond. These findings will also inform curriculum, policy formulation and future research.

### Data sources and search strategy

The initial search was carried out in December 2021 by N.N. The following databases were searched: Science Direct, PubMed, Scopus, ProQuest, Google Scholar, WordCat and MEDLINE. An exhaustive search of these databases was conducted to obtain papers that pertain to simulation as a mode of clinical training in HCPs. The identified papers were included if they were published in English, because of the limited resources available for translation. The papers also needed to focus on simulation as a clinical training method in HCPs. The search terms were as follows: HCPs, simulation-based education, telesimulation, virtual simulation, clinical training, clinical education, continuous professional education, continuous professional development and COVID-19. The same search strategy was used to search Google Scholar and WorldCat to source grey literature such as research reports, dissertations and theses that discuss simulation as a method of clinical training in HCPs. However, no papers were generated through this search. Additionally, as a method of snowball sampling, the reference lists of the identified papers were screened to capture other papers that potentially met the inclusion criteria.

### Citation management

Zotero referencing software was used to save the citations retrieved from the databases. This software allowed for any duplicate citations to be removed. Further duplicate citations, if found, were removed during the title and abstract relevance screening and data characterisation of full articles.

### Eligibility criteria

A two-stage screening to assess the eligibility of the papers identified in the search was employed. Publications that contained the keywords and phrases and (if broadly described) simulation as a mode of clinical training in HCPs were included. To account for advancements in technology as well as to capture contemporary evidence, papers that were published between the years 2011–2021 were included in the review. Publications that described simulation without referring to clinical training and HCPs were excluded from the analysis.

### Title, abstract and full-text relevance screening

For the first level of inspection, only the title citations were reviewed. Thereafter, the abstracts of those citations that were deemed to be eligible for inclusion at the title level were screened. Next, the full articles of those citations that passed the abstract screening were reviewed. If abstracts were not available, the citations were included for the review of the full article. This review was independently conducted by N.N. and B.S. N.N. created a title, abstract and full-text screening spreadsheet. The researchers discussed any discrepancies in the selected papers. Where consensus was not reached, K.K-S. performed the review to make a final decision. There was a high level of agreement between the researchers as the overall kappa was 0.81 (Dohoo, Martin, & Stryhn, 2012).

**FIGURE 1:**
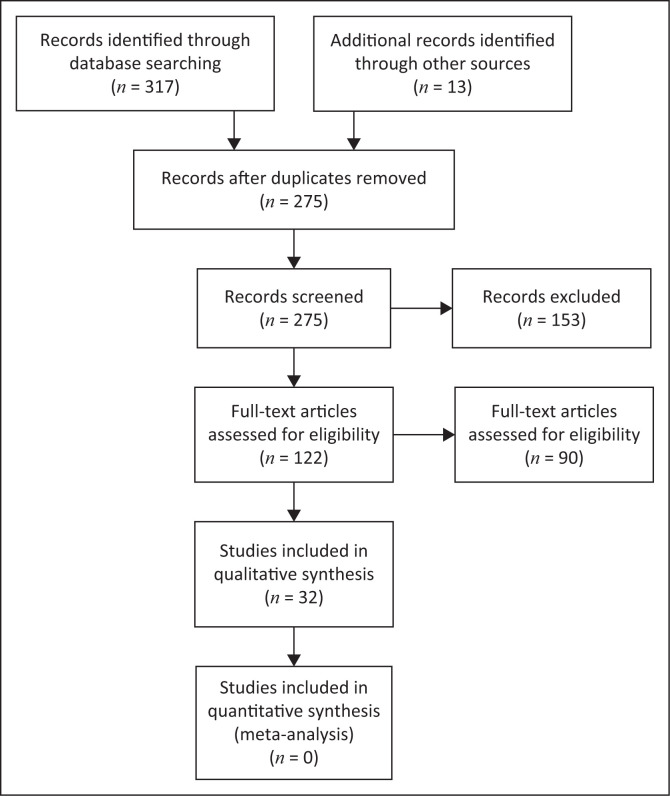
PRISMA flow diagram for the current scoping review which included searches of databases and other sources.

In total, the database search from all databases identified 330 publications. All identified database citations were exported to Zotero, a web-based referencing software. Through Zotero, duplicate studies were identified and removed. After the duplicates were removed, 275 records remained. The 275 records were screened, guided by the search question, and 153 were excluded as not being relevant to the study. One hundred twenty-two articles were then assessed for eligibility; of these, 32 were excluded because they did not meet the inclusion criteria. Consequently, a full-text screening of 90 articles resulted in 32 studies meeting the inclusion criteria and being included in this scoping review. Figure 1 shows a PRISMA flowchart for the literature search, retrieval and inclusion process of this scoping review.

### Data characterisation

Relevant information from the publications that met the inclusion criteria was extracted and noted in the data chart. The data were extracted and charted by N.N., B.S. and M.M. The data charted included the author, year of publication, publication type, publication focus and aims, methodology (study design and sample and data sources, measurement of the outcomes post-simulation training), description of simulation training (preparation for the training, briefing, simulation activity and debriefing), context (country) and results (depicted in Table 1).

**TABLE 1:** Summary of studies included in the scoping review documenting the use of simulation as a clinical training tool.

Author(s) (date)	Publication title	Type of publication	Publication focus and aims	Methodology (including outcome measures of simulation training)	Description of simulation training	Context	Results
						Country	
McGaghie, Issenberg, Cohen, Barsuk and Wayne (2011)	Does simulation-based medical education with deliberate practice yield better results than traditional clinical education? A meta-analytic comparative review of the evidence	Review	Comparing the effectiveness of traditional clinical education toward skill acquisition goals versus simulation-based medical education (SBME) with deliberate practice (DP).	-	-	-	SBME with DP is superior to traditional clinical medical education in achieving specific clinical skill acquisition goals.
Wright et al. (2018)	The influence of a full-time immersive simulation-based clinical placement on physiotherapy student confidence during transition to clinical practice	Research article	Evaluating changes to students’ confidence across a simulation-based placement and clinical competence at the end of their first core clinical placement.	60 physiotherapy students, non-standardised confidence measure and Assessment of Physiotherapy Practice (APP).	Immersive simulation placement; high-fidelity simulated scenarios (incl. simulated patients).	Australia	Improvement confidence: students who completed the simulation-based placement achieved significantly higher APP scores.
Shrestha, Badyal, Shrestha and Shrestha (2020)	*In-situ* simulation-based module to train interns in resuscitation skills during cardiac arrest	Research article	Introducing *in-situ* simulation (ISS)-based resuscitation skills training for medical interns during the event of cardiac arrest.	Prospective quasi-experimental study with mixed research design; 25 medical interns; knowledge-based questionnaire and skill score sheet.	Simulations performed using a primitive manikin or a volunteer and a cardiac rhythm generator.	Nepal	Post-simulation knowledge score was higher; positive correlation between the number of simulations and the skill scores; simulation-safe learning environment for learning; debriefing sessions provided motivation for self-guided learning and allowed for errors to be identified.
Eide, Johansson and Eide (2020)	First-year nursing students’ experiences of simulation involving care of older patients: A descriptive and exploratory study	Research article	Evaluating learning experiences that first-year nursing students had following geriatric patient simulation activity.	Descriptive study; 216 nursing students; survey regarding experiences.	High-fidelity simulated scenarios practised twice; debriefing.	Norway	Applied and integrated theoretical knowledge and practised geriatric nursing in a safe environment and in a practical way under real-world conditions. Better understanding of collaboration. Practising twice allowed for knowledge consolidation.
Johnson, Cannon Mantilla and Cook (2013)	Cricoid pressure training using simulation: A systematic review and meta-analysis	Review	Does cricoid pressure (CP) application improve with simulation training versus with no training, and how is learning retained after CP simulation training?	-	-	-	Simulation had a favourable impact on skills. The CP skill retention only lasted for one week after the simulation.
Silberman, Panzarella and Melzer (2013)	Using human simulation to prepare physical therapy students for acute care clinical practice	Research article	Do specific learning objectives pertaining to the preparation of Doctor of Physical Therapy (DPT) students for acute clinical care describe students’ preparation using this training method?	22 physical therapy students; survey to determine perception of clinical preparedness and benefit of the experience.	Acute care scenario involving a patient following total knee replacement and ready for discharge; high- and low-fidelity manikin, course instructor acted as patient; debriefing.	Brazil	Opportunity to integrate students’ knowledge; better understanding of the expectations of the clinical setting; confidence in communication and management of clinical situations increased.
Korayem and Alboghdadly (2020)	Integrating simulation into advanced pharmacy practice experience curriculum: An innovative approach to training	Research article	Evaluating the impact of integrating simulation-based education into advanced pharmacy practice experience curriculum on students’ learning outcomes, training costs and satisfaction.	57 pharmacy students; clinical competence assessed during rotations.	Simulation-integrated rotations (simulation- and hospital-based rotation) which ran simultaneously; simulations used were of human patient simulators, task trainers and high-fidelity manikins; debriefing.	Saudi Arabia	Higher grades in the simulation-based blocks versus hospital-based blocks; cognitive skills and affect learning outcomes higher in simulation-based block. According to Faculty members, students’ confidence, communication, leadership, and teamwork improved, but students’ patient case presentation delivery still needed improvement. Concurrent running of the rotations resulted in more clinical seats for students and reduced costs.
Zarifsanaiey, Amini and Saadat (2016)	A comparison of educational strategies for the acquisition of nursing students’ performance and critical thinking: Simulation-based training vs. integrated training (simulation and critical thinking strategies)	Research article	To compare the effects of integrated training (simulation and critical thinking strategies) and simulation-based training on the performance level and the critical thinking ability of nursing students.	A pre- and post-test research design; 40 nursing students; California Critical Thinking Skills Test Form B (CCTST-B) to determine critical thinking skills level; Objective Structured Clinical Examination (OSCE) to measure clinical performance.	Experimental group (critical thinking skills strategies and a simulation activity taught by basic instruction, through role-playing and practice on manikins. Control group only engaged in the simulation activity.	Iran	Critical thinking skills and performance level were significantly higher in the experimental group as compared to control group.
Teles, Mendes-Castillo, De Souza Oliveira-Kumakura and Silva (2020)	Clinical simulation in teaching pediatric nursing: Students’ perception	Research article	Undergraduate nursing students’ perceptions about learning to care for the child and family through clinical simulation.	10 nursing care students interviewed.	Engaged with five high-fidelity simulation scenarios relating to paediatric nursing; debriefing.	Brazil	High chance that the scenarios could happen in practice. They felt more secure and confident to make decisions and deal with the situations in practice. Students able to practise before going to the hospital. The scenarios promoted learning of integral care.
Zhang, Cheng, Xu, Luo and Yang (2015)	Clinical simulation training improves the clinical performance of Chinese medical students	Research article	Examining the effectiveness of clinical practice training programs that use simulation.	109 medical interns; OSCE to measure clinical performance.	Simulative clinical training, preclinical skills training complemented with real-life clinical skills prior to internship.	China	Graduate interns who trained with simulations showed learning ability significantly higher than those of interns who only received traditional training. In terms of the clinical skills competition, the results were higher in those who received simulative training than those who did not.
Yang, Li, Liu and Liu (2020)	Effect of vascular simulation training on practice performance in resident: A retrospective cohort study	Research article	Assessing the effect of simulation-based training on improving technical performance and clinical procedures of vascular surgery residents.	95 vascular resident trainees; underwent theoretical and practical assessment.	Experimental group received lectures, engaged in simulation activity at times with direct instruction; practised on part-task trainers. Control group only received lectures.	China	Experimental group had higher scores on the theoretical assessment than control group. Experimental group performed procedures quicker; simulation training could reduce radiation damage of patients and residents.
Cant and Cooper (2017)	The value of simulation-based learning in prelicensure nurse education: A state-of-the-art review and meta-analysis	Review	Current evidence for the effectiveness of simulation as an educational strategy in prelicensure nursing education.	-	-	-	Simulation improves patient safety and students’ clinical competence and awareness. Self-efficacy, cognitive (incl. clinical knowledge), and self-confidence enhanced.
Gunn, Rowntree, Starkey and Nissen (2021)	The use of virtual reality computed tomography simulation within a medical imaging and a radiation therapy undergraduate programme	Research article	Investigating the impact of a computer-based virtual reality (VR) computed tomography (software) simulation on undergraduate medical imaging (MI) and radiation therapy (RT) students’ confidence.	28 MI students; 38 RT students.	Not specified; RT students had an introduction to the virtual reality software.	Australia	MI and RT students’ clinical confidence above average in performing a CT scan. Students preferred to learn CT in the clinical environment; but VR CT simulation software provided some ‘familiarisation’ before proceeding with clinical placement.
Shorey and Ng (2021)	The use of virtual reality simulation amongst nursing students and registered nurses: A systematic review	Review	The use of two variations of virtual worlds desktop virtual reality simulations (dVR) and immersive virtual reality simulations (iVR) as teaching tools for nursing students.	-	-	-	Virtual worlds more effective to teach theoretical knowledge compared to teaching clinical skills and enhancing affective outcomes), but produced worse results compared to the traditional teaching methods for clinical skills, theoretical knowledge and affective outcomes. Advantages of virtual worlds: unlimited in time and space, available for users to practise at any time, less time intensive compared to manikin-based simulations, cost lower than manikin-based simulations. Disadvantages of virtual worlds: technological issues; lack of realism in virtual world.
Kapoor, Kapoor and Badyal (2021)	Simulated patients for competency-based undergraduate medical education post COVID-19: A new normal in India	Review	Need and use of simulated patients (SPs).	-	-	India	SPs used to teach technical, cognitive communication skills and interpersonal skills: SPs can be used with manikins to practise procedural skills on the manikin and communication. Trained SPs provide corrective feedback, allow for reflective learning in a nonthreatening environment.
Offiah et al. (2019)	Evaluation of medical student retention of clinical skills following simulation training	Review	Investigating clinical skills performance following simulation-based medical training in an undergraduate medical curriculum.	-	-	-	Simulation-based mastery learning can improve medical students’ retention of core skills and deliberate practise of clinical skills.
Miles, Friary, Jackson, Sekula and Braakhuis (2016)	Simulation-based dysphagia training: Teaching interprofessional clinical reasoning in a hospital environment	Research article	Evaluating the effectiveness of simulation-based dysphagia training on students’ perceived confidence, knowledge and preparedness.	31 students: 11 dietetics students and 20 speech-language pathology students; post simulation completed clinical vignettes; feedback on perception of the training quality, relevance and clinical applicability; survey on perceived confidence, knowledge and readiness for work, and pre- and post- training.	Three simulated scenarios; high-fidelity manikins, confederates and/or standardised patients. Students worked interprofessional groups.	Australia	Significant increase in student overall scores in clinical vignettes, mostly clinical reasoning. Reported increase in confidence, preparedness and knowledge.
Kononowicz et al. (2019)	Virtual patient simulations in health professions education: Systematic review and meta-analysis by the digital health education collaboration	Review	Evaluating the effectiveness of virtual patients versus traditional education, blended with traditional education, compared with other types of digital education and design variants of virtual patients in health professions education.	-	-	Singapore	Virtual patients to traditional education showed similar results for knowledge and favoured virtual patients for skills.
Hu et al. (2019)	To investigate the effect of simulation-based triage education on third-year nursing students’ self-reported clinical reasoning ability during simulated triage scenarios	Research article	Investigating the effect of simulation-based triage education on third-year nursing students’ self-reported clinical reasoning ability during simulated triage scenarios.	A pretest-posttest research design, 172 nursing students; Nurses Clinical Reasoning Scale (NCRS) utilised to measure pretest and posttest clinical reasoning.	Experimental groups received briefing to triage and simulation (standardised patient and high- and low-fidelity manikins) and self-studied triage content. Control group prepared for triage education; received a lecture. Debriefing on clinical reasoning.	China	After the triage education, significant increase in scores of clinical reasoning ability in all the three groups, average clinical reasoning ability scores in the experimental groups were significantly higher compared to control group.
Choong and Tan (2019)	The role of simulation in burns education	Review	The role of simulation as an important and effective tool for burns education worldwide.	-	-	-	Incorporation of simulations in burns education have improved trauma care and survival of victims. Use of manikins enhances technical, procedural skills, confidence levels; non-technical skill; moulage contributed to positive training experience, encourages to treat standardised patients as real burns victims.
De Ponti et al. (2020)	Pregraduation medical training including virtual reality during COVID-19 pandemic: A report on students’ perceptions	Research article	Assess medical students’ perception on fully online training, including simulated clinical scenarios during COVID-19 pandemic.	115 medical students; questionnaire to rate training quality.	Briefing followed by simulated clinical scenarios conducted online; debriefing.	Italy	77% considered virtual reality training realistic for the initial clinical assessment, 94% for the diagnostic activity and 81% for the treatment options; 84% considered the future use of this virtual reality training useful in addition to traditional clinical training; no one recommended the standalone use of this virtual reality; 28% participants found the online access difficult due to technical issues.
Grabowski et al. (2021)	A pilot study to evaluate the effect of classroom-based high-fidelity simulation on midwifery students’ self-efficacy in clinical lactation and perceived translation of skills to the care of the breastfeeding mother-infant dyad	Research article	Evaluate the impact of a classroom-based breast-feeding simulation on nurse-midwifery students’ self-efficacy in lactation skills.	A pilot study using a prospective cohort study design. Study sample: 9 nurse-midwifery students. Self-efficacy surveys used to measure self-efficacy.	Two high-fidelity simulation-based workshops that focused on competencies for midwives and lactation professionals.	United States of America	Increase in perceived self-efficacy skills in basic and advanced clinical lactation skills after completing both workshops.
Vermeulen et al. (2017)	The experiences of last-year student midwives with high-fidelity perinatal simulation training: A qualitative descriptive study	Research Article	Experiences of last-year student midwives with high-fidelity perinatal simulation training.	Descriptive research design; 13 midwives; focus groups.	High-fidelity perinatal simulation sessions.	Australia	High-fidelity perinatal simulation training to be a positive learning method that increased students’ competence and confidence.
Büyük et al. (2021)	Use of simulation to teach in the operating room: Don’t let the COVID-19 pandemic to interrupt education: An observational clinical trial	Research article	Evaluating the difference between the pre- and post-simulation knowledge test scores to evaluate the effectiveness of simulation training. Examining if it creates any anxiety on participants.	Study sample, anaesthesiology and intensive care residents; true/false questions and State-Trait Anxiety Inventory used to assess knowledge and anxiety before and after activity, respectively.	*In-situ* simulation activity using the Resusci Anne manikin, debriefing.	Turkey	Junior residents received significantly higher scores in post-training theoretical tests compared to their pretraining scores. There was no difference between pretest and posttest scores of seniors. Pre- and post-anxiety inventory scores were nearly the same and both were in the moderate group.
Mileder, Bereiter and Wegscheider (2021)	Telesimulation as a modality for neonatal resuscitation training	Research article	Investigating the feasibility and effectiveness of telesimulation for neonatal resuscitation training.	Prospective observational pilot study; 9 medical students and nine neonatal nurses; questionnaire regarding their knowledge of neonatal resuscitation training.	Simulated session conducted remotely broadcasted from a clinical skills training centre. Students had items for the activity, including a low-fidelity manikin.	Austria	Telesimulation enjoyable, gained knowledge, educational methodology suitable for neonatal resuscitation training, still preferred traditional face-to-face instruction due to potential technical issues, training logistics and the quality of supervision.
Hernández-Padilla, Granero-Molina, Márquez-Hernández, Cortés-Rodríguez and Fernández-Sola (2016)	Effects of a simulation-based workshop on nursing students’ competence in arterial puncture	Research article	Evaluating if a short simulation-based workshop in radial artery puncture improves nursing students’ competence to the extent that patients’ safety is not compromised.	Pretest-posttest design; 86 undergraduate nursing students; core clinical competencies arterial blood gas (ABG) analysis in simulation activity.	Briefing activity; watched a video on and provided with examples of ABG analysis; conducted ABG analysis independently.	Spain	Posttest scores for knowledge, skills, and self-efficacy were higher than the pretest scores; 61.1% of the participants showed the level of competence required to safely practise radial artery puncture on a live patient.
Roberts et al. (2019)	Simulation to replace clinical hours in nursing: A metanarrative review	Review	Review of published literature on the replacement of part of the clinical placement hours with simulation in undergraduate nursing education and its feasibility.	-	-	Australia	No significant differences between clinical experience and simulation when simulation replaces up to 50% of clinical hours. Comparable student engagement, educational outcomes and student proficiency with simulation and traditional clinical practice.
Boudiche et al. (2020)	Simulation training for continuing professional development of nurses in cardiology and cardiovascular surgery	Research article	Clarifying the interest of simulation learning in terms of continuing professional development involving nurses in the management of cardiac arrest.	Comparative prospective observational study; 32 nurses; skill evolution sheet to assess basic life support and manual defibrillation.	Provided with theory on basic and advanced life supports; provided basic life support on a low-fidelity manikin and manual defibrillation on a high-fidelity manikin.	Tunisia	Significant improvement was noted after the simulation session in terms of diagnosing cardiac arrest and providing basic life support and manual defibrillation.
Warren, Luctkar-Flude, Godfrey and Lukewich (2016)	A systematic review of the effectiveness of simulation-based education on satisfaction and learning outcomes in nurse practitioner programs	Review	Included studies that explored any learner outcome related to high-fidelity simulation (HFS) in nurse practitioner (NP) programmes	-	-	-	Nursing students perceived enhancement of their critical thinking skills, evidence-based practice and knowledge and ability to function in the clinical setting when using HFS. Significant increase in knowledge and technical skills post-engagement with HFS and compared to those who received traditional education. Higher clinical compared to web-based education. High-fidelity simulation has positive effects on NP student confidence and self-efficacy.
Sun, Pan, Li and Gan (2017)	Airway management education: simulation-based training versus non-simulation-based training: A systematic review and meta-analyses	Review	To evaluate all published evidence comparing the effectiveness of simulation-based training (SBT) for airway management versus non-simulation-based training (NSBT).	-	-	-	SBT-improvement in performance behaviours but did not transfer into increased success rate in clinical setting. No significant difference in success rate of procedure completion on patients in both groups; no patients treated by students in both groups suffered any significant adverse effects. Skills decay significantly in both groups. Confidence scores were higher in SBT group versus NSBT group.
Datta, Upadhyay and Jaideep (2012)	Simulation and its role in medical education	Review	No specified.	-	-	-	Simulation can complement traditional clinically based training.

HFS, high-fidelity simulation; SBME, simulation-based medical education; DP, deliberate practice; APP, Assessment of Physiotherapy Practice; CP, cricoid pressure; DPT, Doctor of Physical Therapy; CCTST-B, California Critical Thinking Skills Test Form B; OSCE, Objective Structured Clinical Examination; VR, virtual reality; MI, medical imaging; RT, radiation therapy; dVR, desktop virtual reality; iVR, immersive virtual reality; SPs, simulated patients; NCRS, Nurses Clinical Reasoning Scale; HFS, high-fidelity simulation; NP, nurse practitioner; SBT, simulation-based training.

### Data summary and synthesis

The data were compiled in a single spreadsheet and imported into Microsoft Excel 2010 (Microsoft Corporation, Redmond, WA) for descriptive analysis, where thematic analysis was conducted by N.N., K.K-S. and N.M. to analyse the data extracted.

## Results and discussion

As depicted in Table 1, 32 papers were included in the final analysis. Of these papers, 21 presented empirical data, whilst 11 were reviews. Of the total sample of studies reviewed, only four were conducted in LMICs, with the rest from high-income countries (HICs). Additionally, 28 of the studies were conducted prior to the COVID-19 pandemic, with only four conducted during COVID-19.

Nuanced analysis of the studies, as reflected in Table 1, revealed that the use of simulation has been investigated in basic medical (e.g. McGaghie et al., 2011; Zhang et al., 2015), surgery (Yang et al., 2020), nursing (e.g. Eide et al., 2020; Teles et al., 2020), radiography (Gunn et al., 2021), physiotherapy (Silberman et al., 2013; Wright et al., 2018) and pharmacy (Korayem & Alboghdadly, 2020) education. The review revealed an obvious gap in evidence regarding the utility of simulation in the clinical training of SLP&A professions, as none of the studies considered in this review explored the use of simulation in audiology, with only one study that explored the utility of simulation in speech-language pathology (Miles et al., 2016). However, this one study focused on simulation usage in teaching interprofessional clinical reasoning in speech-language therapy and dietetic students, and not on core clinical skills in SLP.

Of the studies reviewed, the use of simulation as a mode of clinical training has been explored mostly for undergraduate and postgraduate HCPs students. Very few studies have described its use as a method of continuous professional education in qualified HCPs (e.g. Boudiche et al., 2020; Rossler, Hardin, Hernandez-Leveille, & Wright, 2018), and this is an important area of training for SLP&A in South Africa as continued professional development, particularly with the rapid technological advancements in SLP&A, is a mandated regulation by the Health Professions Council of South Africa (HPCSA). This is despite Rossler et al. (2018) and Boudiche et al. (2020) indicating the effectiveness of simulation training as a continuous professional development tool and HCPs perceiving it to be a valuable clinical training method to enhance their clinical practice.

Thematic analysis of the papers reviewed revealed three themes. These themes that emerged from the data were as follows: (1) face-to-face simulations as a mode of clinical training, (2) virtual reality simulation (VRS) and telesimulation as modes of clinical training and (3) simulation as a complementary mode of clinical training. The rest of the scoping review findings will be presented under these three themes.

### Face-to-face simulation as a mode of clinical training

Most of the studies included in this scoping review discussed face-to-face simulations, which comprised at least a simulation scenario and debriefing sessions (e.g. De Ponti et al., 2020; Hu et al., 2019; Shrestha et al., 2020). Although the purpose of conducting debriefing sessions has not been explicitly highlighted in the studies, a finding from one of the studies has alluded to the practice of debriefing as allowing students to reflect on their performance in the simulation scenario and thus improve on their weaker areas before they begin with their clinical placements (Shrestha et al., 2020). The scenarios involved interactions with a variety of simulators ranging from low-fidelity (part-task trainers and low-fidelity manikins) to high-fidelity simulators (high-fidelity manikins and simulated or standardised patients).

In LMICs, it seems that when simulation is used as a mode of clinical training, face-to-face simulations that incorporate low-fidelity simulators and students simulating patients are used (Boudiche et al., 2020; Shrestha et al., 2020; Teles et al., 2020). The authors of this scoping review query if this may be because of high-fidelity simulators being costly. The authors also found it interesting that virtual simulations were never used as a means of training in these countries, especially because virtual training platforms are thought to be feasible for HCPs who work in resource-constrained countries (Asangansi & Braa, 2010). Furthermore, the Medical Council of India (2019) is reforming medical education for it to be competency-based, so they have recommended the incorporation of face-to-face simulations in medical education. This recommendation motivated a review conducted by Kapoor et al. (2021) on the utility of simulated patients as a teaching tool.

The findings from the review revealed that incorporating simulated patients into medical education may allow medical students to achieve the core clinical competencies. Face-to-face simulations improved nurse-midwifery, nursing and physiotherapy students’ self-efficacy and confidence (e.g. Silberman et al., 2013; Teles et al., 2020; Wright et al., 2018). It also enhanced nursing and physiotherapy students’ knowledge in practically applying and integrating their knowledge in a safe environment (Eide et al., 2020; Offiah et al., 2019). It also enhanced their understanding of the need for collaboration with other HCPs (Eide et al., 2020). Face-to-face simulation also vastly improved nurses’ and physiotherapists’ clinical task and non-clinical task skills, particularly when high fidelity simulations were used (Kapoor et al., 2021; Silberman et al., 2013; Vermeulen et al., 2017). This may be a result of high-fidelity manikins being able to be programmed to produce physical responses and simulated or standardised patients being able to replicate patient encounters (Kapoor et al., 2021). Utilising high-fidelity simulations does not only allow for the consolidation of knowledge and skills but also seems to provide nursing students with the opportunity to practise providing holistic patient care (Teles et al., 2020). This mode of training may explain why nurses felt prepared to work with real patients and why simulation is considered to improve patient outcomes. Additionally, because face-to-face simulation training enhanced vascular residents’ skills in performing surgery, they were able to perform cerebral vascular angiography procedures quicker, which meant that the potential radiation damage caused by this procedure is reduced in residents and patients (Yang et al., 2020).

Although face-to-face simulations have been shown to improve students’ clinical competency, there are concerns that skill retention after solely face-to-face simulation training is short (Johnson et al., 2013; Offiah et al., 2019). One way to enhance medical and nursing students’ skill retention is for them to repeat the simulation activities (Eide et al., 2020; Offiah et al., 2019). Additionally, studies by Zarifsanaiey et al. (2016), Offiah et al. (2019) and Korayem et al. (2020), as well as those incorporated in McGaghie et al.’s (2011) review, have shown that when face-to-face simulations are supplemented or integrated with other teaching or clinical training methods, pharmacy, medical and nursing students’ knowledge and clinical task skills and retention thereof is further enhanced. Furthermore, to allow for the continuation of clinical training of HCPs in the COVID-19 era, some programmes, like anaesthesiology programmes, implemented face-to-face simulations as a mode of clinical training (Büyük et al., 2021).

### Virtual reality simulation and telesimulation as modes of clinical training

Face-to-face simulation training was not always possible because of the social distancing regulations implemented during the COVID-19 pandemic. Therefore, medical programmes used telesimulation or VRS to train medical students (De Ponti et al., 2020; Mileder et al., 2012). However, VRS have been used prior to the pandemic. A review by Shorey and Ng (2021) found that VRS, namely, desktop virtual reality and immersive VRS were less costly and less time-intensive than face-to-face simulations that involve manikins. Virtual reality simulation is also not limited by time and space constraints, and students can practise any time. However, technical issues may be incurred when using VRS and telesimulation, which may frustrate students. Furthermore, these types of simulations may lack authentic realism (Gunn et al., 2021).

This lack of authentic realism may explain why HCP students’ clinical tasks skills still need to be enhanced in traditional clinical training. This point may be reinforced by the findings of Gunn et al. (2021) and De Ponti et al. (2020), who found that medical imaging and radiation therapy students and medical students, respectively, preferred to learn procedures in traditional clinical placements. However, these students acknowledge that VRS may be used to prepare them for their clinical placements and can be an effective clinical training tool when supplemented with traditional clinical training. However when virtual patients are incorporated in VRS HCP, students’ skills seem to be enhanced (Kononowicz et al., 2019). Similarly, telesimulation may allow students to gain knowledge and practise their clinical task skills, but face-to-face simulation is still a preferable means to acquire skills (Mileder et al., 2012). Therefore, although face-to-face simulation training may result in better learning outcomes compared to VRS and telesimulation, VRS and telesimulation have not shown to produce worse learning outcomes compared to face-to-face simulation (Mileder et al., 2021; Shorey & Ng, 2021).

### Simulation as a complementary mode of clinical training

A review conducted by Roberts et al. (2019) documented that the clinical core competencies achieved by nursing students whose portion of traditional clinical training hours was replaced by face-to-face simulation were the same as those who only received traditional clinical training. Additionally, a study conducted by Korayem and Alboghdadly (2020) found that integrating face-to-face simulation with traditional clinical training and running these activities simultaneously allowed for the increase in student numbers to be accommodated because of the increase in clinical seats for students. In contexts where simulations have not been widely used as a mode of clinical training, such as South Africa, the current authors believe that the use of simulation as a complementary mode should be deliberated on for gradual and safe introduction of this mode of training in clinical training programmes. This would require more collaborative deliberations between training institutions and the regulating body, the HPCSA, so that such training can form part of the regulations as well as clinical hours for registration, as these changes have already begun during the 2020–2021 period where students were allowed to claim 20% of hours via simulation. Additionally, it is particularly important to consider simulations at least as a complementary mode of clinical training when planning clinical training for South African SLP&As, because ASHA (n.d.) recognises clinical simulation as an alternative means of traditional clinical training for American SLP&A students; meanwhile, the CFCC (2016) has allowed American SLP&A students to obtain 75 hours of direct clinical contact training through the use of simulations.

## Conclusion

The current scoping review provides evidence that simulation is an effective method of clinical training in basic and specialised medical education, nursing, radiography, pharmacy and physiotherapy as it enhances the clinical competence of HCP students in these programmes. Therefore, it may equip students with knowledge, skills and attitudes required to interact with patients that they encounter in their clinical placements and independent practice. Simulation, particularly VRS and telesimulation, can be used as a mode of clinical training to allow for the continuation of clinical training of HCP students and practitioners despite the COVID-19 restrictions. However, simulation cannot replace traditional clinical training but may be combined with traditional clinical training in a hybrid mode – which may further enhance HCPs students’ clinical competence.

As alluded to earlier, because of resource constraints, the large number of students enrolled in SLP&A programmes cannot be sufficiently accommodated for particularly in terms of clinical placements. Therefore, the current authors recommend deliberations around utilising simulations prior to traditional clinical placements, as a way of hastening acquisition of core clinical competencies, thus allowing students more opportunities to practise (both depth and breadth of the scope of the professions). The improved preparedness may also lessen the workload of clinical educators, whilst enhancing skills for independent safe practice (Cant & Cooper, 2017). Furthermore, if simulation is integrated within traditional clinical training and these modes of training run concurrently, clinical placements may be able to accommodate the increased numbers of students (Korayem & Alboghdadly, 2020). These suggestions, however, require regulatory approval as they have implications for the number of credits for courses as mandated by the South African Qualifications Authority (SAQA) as well as clinical hours required for registration of HCPs by the HPCSA. Furthermore, when considering the use of simulations as a mode of clinical training for South African SLP&A students, reference should be made to guidelines that have outlined the standards of using simulation as a mode of clinical training for SLP&A students, such as the ASHA (n.d.) guidelines.

The studies included in this scoping review have mostly provided insight into the use of simulations for clinical training in HCPs other than SLP&A. However, the studies may still support the consideration for using simulations in SLP&A clinical training, as clinical training in all HCPs is based on the same core clinical competencies (Medical and Dental Professions Board, 2016). When considering simulation as a mode of clinical training, clinical educators’ competency in facilitating the activities needs to be considered. Inadequate skills in facilitating simulation activities will result in simulation training being ineffective (Krishnan, Keloth, & Ubedulla, 2017). Therefore, clinical educators facilitating these sessions need to receive training and mentoring regarding using simulators, creating and facilitating simulation activities and integrating simulations into the curriculum so that students can achieve the core clinical competencies (Koukourikos et al., 2019). Whilst simulation in HCP education aims to mimic real-life clinic scenarios, the degree of realism depends on the fidelity of the simulator, the setting and the simulation activity (Kapoor et al., 2021; Koukourikos et al., 2019). Therefore, the degree of realism needs to be accounted for when planning to use simulation as a mode of clinical training. Healthcare professions students seem to acknowledge the benefit of simulations being incorporated into their clinical training, but they still prefer that traditional clinical training be used at least in addition to simulations (e.g. Gunn et al., 2021; Mileder et al., 2021).

Therefore, research on the utility of simulation and how it can be incorporated in SLP&A is required. Specifically for LMICs, whilst simulation has proven to be cost-effective and a responsive mode of training HCPs during public health emergencies as evidenced during COVID-19 and beyond, there are contextual realities that cannot be ignored. These include resources that are not readily available because of finances and poor infrastructure (Khoza-Shangase, 2021), as well as a lag in the uptake of technological advances as part of clinical service provision and training (Khoza-Shangase et al., 2021). Furthermore, LMICs have a large rural population, limited health education and limited access to technology resources within poorly performing economies (Muttiah, McNaughton, & Drager, 2016). Therefore, whilst remote learning, and by extension, telesimulation is effective in HICs, its success in LMICs requires supportive measures such as supplements to poor internet bandwidth, strategies to address shortage of trained personnel (Pillay et al., 2020) and initiatives to improve limited computer skills for users (Khoza-Shangase et al., 2021).
